# Current Status of Gene Therapy Research in Polyglutamine Spinocerebellar Ataxias

**DOI:** 10.3390/ijms22084249

**Published:** 2021-04-19

**Authors:** Ricardo Afonso-Reis, Inês T. Afonso, Clévio Nóbrega

**Affiliations:** 1 ABC-RI, Algarve Biomedical Center Research Institute, Universidade do Algarve, 8005-139 Faro, Portugal; reisricardo12@hotmail.com (R.A.-R.); inesta.28@gmail.com (I.T.A.); 2 Faculdade de Medicina e Ciências Biomédicas, Universidade do Algarve, 8005-139 Faro, Portugal; 3 Champalimaud Research Program, Champalimaud Center for the Unknown, 1400-038 Lisbon, Portugal

**Keywords:** polyglutamine disorders, spinocerebellar ataxia, gene therapy, gene augmentation, gene silencing, gene editing

## Abstract

Polyglutamine spinocerebellar ataxias (PolyQ SCAs) are a group of 6 rare autosomal dominant diseases, which arise from an abnormal CAG repeat expansion in the coding region of their causative gene. These neurodegenerative ataxic disorders are characterized by progressive cerebellar degeneration, which translates into progressive ataxia, the main clinical feature, often accompanied by oculomotor deficits and dysarthria. Currently, PolyQ SCAs treatment is limited only to symptomatic mitigation, and no therapy is available to stop or delay the disease progression, which culminates with death. Over the last years, many promising gene therapy approaches were investigated in preclinical studies and could lead to a future treatment to stop or delay the disease development. Here, we summed up the most promising of these therapies, categorizing them in gene augmentation therapy, gene silencing strategies, and gene edition approaches. While several of the reviewed strategies are promising, there is still a gap from the preclinical results obtained and their translation to clinical studies. However, there is an increase in the number of approved gene therapies, as well as a constant development in their safety and efficacy profiles. Thus, it is expected that in a near future some of the promising strategies reviewed here could be tested in a clinical setting and if successful provide hope for SCAs patients.

## 1. Introduction

**Figure 1:**
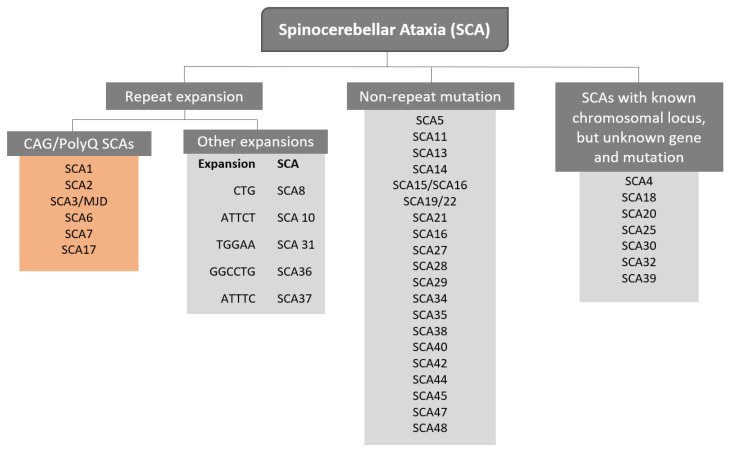
Representation of genetic classification of spinocerebellar ataxias.

The term Spinocerebellar refers to the spinal cord and cerebellum, and ataxia means “absence of coordination”. So, Spinocerebellar Ataxias (SCAs) are a group of degenerative diseases of the nervous system in which progressive ataxia occurs [1]. Until now, there are more than 40 genetically different SCA subtypes identified, which include inherited autosomal recessive cerebellar ataxias, autosomal dominant spinocerebellar ataxias, and X-linked ataxias [2,3,4]. The nomenclature is given by the term SCA followed by a number, representative of the chronological order of the causative gene or disease locus discovery. The latest discovered subtype is SCA 48 [5], however, some numbers are vacant, as some subtypes overlap or share the same muted gene e.g., SCA15 and SCA16, and SCA19 and SCA22 [6]. SCAs are categorized into two subgroups according to the causative genetic origin: the non-repeat mutations and repeat expansions SCAs (Figure 1). SCAs are rare inherited diseases affecting approximately 1–5:100,000 persons worldwide [7]. The most common is SCA3/MJD followed by SCA2 and SCA6, respectively [8]. The three countries where SCAs are more prevalent are: Portugal with the highest rates in population registering 5.6:100,000 [8], followed by Norway registering rates of 4.2:100,000 [9], and Japan registering rates of 5:100,000 [10]. In this review, we are going to focus specifically on the autosomal dominant SCAs with CAG expansion, also known as Polyglutamine (PolyQ) SCAs.

PolyQ SCAs are caused by an abnormal CAG trinucleotide repeat expansion within the coding region of the causative gene that encodes for the glutamine amino acids, generating an expanded polyglutamine protein. The repeat expansion size varies from individuals, being related to the disease severity, and the age onset, that is, individuals with higher CAG repetitions display an early onset of the symptoms, which are also more severe [11]. PolyQ SCAs are also characterized by the anticipation of the age onset, explained by intergenerational instability biased towards expansions. Additionally, somatic mosaicism was also reported as being associated with the age of onset and the severity of the symptoms [12]. The disease onset is usually in the fourth decade of life, with a life expectancy that can vary between 10–15 years [13]. The first symptoms are usually gait ataxia, frequently followed by limb incoordination, speech disturbance, and oculomotor abnormalities [14] according to the SCA subtype. Although the symptomatology is similar between PolyQ SCAs, different repeat size and genes are responsible for the SCA subtype (Table 1).

### Therapeutic Advances

No cure for PolyQ SCAs is known, however several strategies to slow the disease progression and prolong lifespan were proposed and investigated [15]. The exact pathogenic molecular mechanism underlying PolyQ SCAs has not been completely understood. However, the general pathophysiological features present in PolyQ SCAs patients, arise from histological and ultrastructural observations, such as the occurrence of intraneuronal inclusions of unfolded proteins [16], RNA toxicity [17], mitochondrial dysfunction [18], and channelopathies [19,20]. The understanding of the full mechanisms underlying the molecular pathogenesis is crucial for the development of therapies targeting those different aspects of the disease. In fact, several of the gene therapies were developed to target different pathways that are implicated in the disease pathogenesis. In this line, the strategies proposed for polyQ SCA treatment comprise the targeting of (i) mutant RNA, (ii) autophagy and ubiquitin-protease system, (iii) neuroprotective pathways, (iv) transcriptional dysregulation, (v) post-translational modifications, (vi) mutant protein cleavage, (vii) mutant protein aggregation, (viii) inflammation, (ix) excitotoxicity, (x) endoplasmic reticulum stress, (xi) calcium homeostasis, (xii) mitochondrial dysfunction and (xiii) synaptic dysfunction [21,22] (Table 2).

Several pharmacological strategies were investigated targeting different points of the molecular pathogenesis, aiming for example to potentiate protein clearance mechanisms, or to inhibit the toxic protein fragments generation [13,23]. An example is cordycepin, which was tested in a transgenic SCA3/MJD mouse model. The results indicate a significant reduction in the mutant ataxin-3 levels and neuropathological abnormalities, as well as an amelioration of the motor and neuropathological deficits [24]. Other drugs such as trehalose [25] and ibuprofen [26] have also demonstrated to be neuroprotective therapy approaches in this disease.

Non-pharmaceutical strategies used the administration of mesenchymal stem cells (MSCs) [27,28] and stem cells [29] as a strategy to decrease PolyQ SCAs progression. A study using neural precursor cells derived from a transgenic SCA1 mice model demonstrated recovery of the motor behavior and morphological improvement of Purkinje cells when injected in the cerebellar white matter of SCA1 mice [30]. A similar study injected cerebellar neural stem cells in adult transgenic SCA3/MJD mice and likewise, the motor behavior impairments were significantly reduced, as well as the number of mutant *ATXN3* aggregates, Purkinje cells loss, and shrinkage of cellular layers [31].

Other recent and advanced non-pharmaceutical strategies to PolyQ SCAs emerge from the use of different gene therapy strategies, namely gene augmentation, gene silencing, and gene editing. These molecular therapies have the ability to delay the disease progression or potentially cure terminal or severely disabling conditions. Due to the generation of cell specific delivery systems packed with information-rich gene-based cassettes, is possible to mediate persistent, stable and robust transgene expression [32]. The recent approval of gene therapy agents by the European authorities increase the interest of researchers in these strategies, as well as hope for many patients suffering for incurable diseases [33]. This review led us to expand our understanding on the current investigation targets for this disease group and highlight the most promising strategies. However, as discussed in the future perspectives section, until now none of these strategies reached clinical trials and therefore research must continue aiming to deliver one of these strategies to patients in the future.

## 2. Gene Therapy Augmentation Strategies

The gene therapy augmentation strategy is a simple and straightforward method where a new protein-coding gene is added to a target cell or organ [34]. This method is particularly suitable for monogenic recessive diseases, where only adding one copy of the normal allele is enough for phenotype reversion, and ultimately cure the disease. This specific strategy can also be named as gene replacement therapy, in the context where the dysfunctional or the lack of a protein is overcomed by adding the correct version of the coding gene. However, for monogenic dominant or complex diseases, as in case of PolyQ SCAs, this approach is not sufficient, and therefore other strategies, such as, gene silencing or gene editing would have a better outcome in reverting the disease phenotype [34]. While this is true, several studies have shown that gene addition therapy could be useful in the context of PolyQ SCAs. As mentioned, several pathways and molecules are dysregulated and implicated in PolyQ SCAs pathogenesis. Thus, several studies investigated the delivery of protein-coding genes that encode for growth factors, neuronal homeostasis, or autophagy-activating proteins, such as the insulin-like growth factor [35], DNAJ proteins [36], beclin-1 [37] respectively, as a strategy to counteract the pathology phenotype. These strategies have different mechanisms of actions and target different aspects of the disease pathogenesis, nevertheless they all aim to ameliorate neuronal homeostasis and through this mitigate the disease phenotype. This method aims mostly to delay the disease progression by ameliorating neuronal homeostasis. Considering this, we will review gene augmentation studies aiming to activate autophagy or to have a neuroprotective impact.

### 2.1. Strategies Activating Autophagy

Eukaryotic cells have two main mechanisms to degrade misfolded proteins: the autophagy and the ubiquitin-proteosome system [18]. In a simplistic way, the autophagy process starts with the engulfment of intracytoplasmic proteins and organelles into a double-membrane vesicle forming the autophagosome. Afterward, the fusion between the lysosome and the autophagosome occurs, leading to degradation of the autophagosome content [38]. Important studies in vivo have shown that the knockout of essential autophagy proteins and the consequent autophagy impairment led to a neurodegeneration phenotype [39,40]. In PolyQ SCAs, several studies established autophagy dysregulation as a common feature in the molecular pathogenesis of these diseases and a preferential target for therapeutic development (Table 3). For example, polyQ protein cytosolic aggregates co-localize with important autophagy proteins, suggesting that these components are sequestrated to aggregates, therefore preventing their normal function and impairing autophagy functioning [41]. Although autophagy activation seems like a promising strategy, it would be more efficient for PolyQ SCAs with cytoplasmic aggregates localization, such as SCA2 and SCA6, or to SCA3/MJD that has both intranuclear and cytosolic locations [37].

An investigation aimed to reinstate the cholesterol 24-hydroxylase (CYP46A1) in SCA3/MJD disease models, which were shown to be downregulated [42]. This study showed that CYP46A1 expression is able to specifically upregulate autophagy, which was shown to be dysfunctional in SCA3/MJD [43]. CYP46A1 is a cholesterol efflux enzyme that is involved in the brain cholesterol metabolism and its levels are significantly reduced in the brain of SCA3/MJD patients and also in mouse models. The re-establishment of CYP46A1 showed a reduction in ataxin-3 aggregates accumulation, alleviated disease-associated neuronal abnormalities, and improved motor deficits [42].

Another study, analyzed brain tissue from SCA3/MJD patients and found an abnormal expression of several autophagic markers, a dysfunctional accumulation of autophagosomes and decreased levels of beclin-1, a protein involved in autophagy [43]. Using a lentiviral vector encoding for beclin-1, the researchers overexpressed beclin-1 in two different mouse models of SCA3/MJD [44]. The results indicated that when injected in an early stage of the disease, beclin-1 overexpression could prevent the neuropathology and behavior deficits. However, when injected in a late stage of the disease, beclin-1 only partially block the disease progression [44].

Following the same line, a study identified that Homer-3 expression, a Purkinje-enriched scaffold protein that regulates neuronal activity, is impaired in SCA1 as a consequence of reduced mTORC1 signaling [45]. mTORC1 is an autophagy regulator, that when inhibited exacerbates the disease pathology. In this study, a mouse model of SCA1 was injected with adeno-associated virus (AAV) vector carrying Homer-3. The results showed that Homer-3 overexpression ameliorated climbing fibers deficits, reduced spine loss and enhanced mTORC1 signaling in Purkinje cells [45]. However, no motor evaluation of the animals upon Homer-3 overexpression was performed.

### 2.2. Neuroprotective Strategies

The PolyQ SCAs expanded protein and aggregates accumulation, interfere with the cellular homeostasis thus contributing to neuronal degeneration [46]. As such, several gene therapy strategies aimed to activate neuroprotective strategies, in order to protect neuronal homeostasis.

In the context of SCA1, a study investigated the overexpression of human ataxin-1-like in an effort to compete with mutant human ataxin-1 [47]. The results showed that human ataxin-1-like overexpression improved motor coordination in SCA1 mouse model and led to an improvement of neuronal function. Thus, authors suggested that ataxin-1-like overexpression is a promising candidate for pre-clinical experiments [47]. Similarly, another study was conducted with the aim of increasing the expression of normal ataxin-3 in a lentiviral SCA3/MJD model. However, authors found that wild-type ataxin-3 overexpression did not show protection against the SCA3/MJD pathology, which could be explained by the interaction of mutant ataxin-3 with the wild-type form that promotes its translocation to the nucleus [48].

Another study showed that ataxin-2 levels, a translation regulatory protein, is reduced in samples from SCA3/MJD patients and in animal models of disease [49]. Therefore, the re-establishment of ataxin-2 levels was promoted using lentiviral vectors in different cellular and animal models of the disease. The results showed that the re-establishment of ataxin-2 levels reduced mutant ataxin-3 levels, the number of intraneuronal aggregates, and mitigates motor deficits, suggesting a possible neuroprotective impact of ataxin-2 in the context of SCA3/MJD [49].

The proteolytic cleavage of mutant ataxin-3 leads to the generation of cytotoxic aggregates of protein products, resulting in large intracellular inclusions that many associate with the SCA3/MJD pathogenesis. A family of proteins involved in the proteolytic cleavage are the calpains, which are known to be involved in ataxin-3 cleavage [17,50]. A study, overexpressed endogenous calpastatin, a calpain-specific inhibitor, in a lentiviral mouse model of SCA3/MJD [51]. Calpastatin overexpression prevented mutant Ataxin-3 cleavage, its translocation to the nucleus and the formation of nuclear aggregates [51].

Also in SCA3/MJD, it was identified a new guanosine triphosphatase named CRAG (collapsin response mediator protein (CRMP)-associated molecule (CRAM[CRMP-5])-associated GTPase) that facilitates the PolyQ aggregates degradation through the ubiquitin-protease pathway [52]. In a transgenic mouse model of SCA3/MJD the administration of CRAG mediated by lentiviral vectors showed a clearance of PolyQ aggregates and an improvement in the mice ataxic phenotype. Also, Purkinje cells misarrangement and disorientation in the CRAG-treated transgenic mice was ameliorated compared to control animals [52].

A study used neuropeptide Y (NPY), as a strategy to mitigate SCA3/MJD phenotype due to its features as an inhibitor of cell death, autophagy stimulator, anti-inflammatory effect, and increased trophic support [53]. Transgenic SCA3/MJD mice were injected with AAV vectors overexpressing NPY. The results showed that NPY overexpression rescued motor and balance impairments and reduced the number of mutant ataxin-3 aggregates, prevented microglial immunoreactivity and significantly reduced proinflammatory cytokine Il6 mRNA levels. Overall, these results showed a mitigation of the disease associated neuropathology and motor and balance-related deficits upon NPY expression [53].

The autosomal dominant trait of PolyQ SCAs contributes to the limited research and development of gene augmentation strategies, being mainly centered in SCA1 and SCA3/MJD. Some of the results from the above-mentioned studies demonstrate promising results using gene augmentation as a strategy to delay disease progression for SCA1 and SCA3, as they were able to reduce important neuropathological abnormalities. Even though none of these strategies could represent a definitive cure for PolyQ SCAs, these studies aimed to restore dysfunctions underlying the pathogenesis and therefore mitigate the disease phenotype. The strategies here presented might be extendable to the other PolyQ SCAs, as they share similar mechanisms of pathogenesis. However, to proceed with these strategies to clinical trials more data is necessary, such as testing in larger mammal models, i.e., non-human primates, to address the beneficial effects and importantly to access the safety profile of delivering these genes.

**Table 3:** Selected gene augmentation strategies studies in polyglutamine spinocerebellar ataxias.

Disease	Molecular Target	Gene Delivery System	Strategy	References
SCA1	Homer-3	AAV vectors	Autophagy	[45]
	Ataxin-1 like	AAV vectors	Neuroprotection	[47]
SCA3	CYP46A1	AAV vectors	Autophagy	[42]
	Beclin-1	Lentiviral vector	Autophagy	[44]
	Calpastatin	AAV vectors	Proteolytic cleavage	[51]
	Wild-type ataxin-3	Lentiviral vector	Neuroprotection	[48]
	Ataxin-2	Lentiviral vector	Neuroprotection	[49]
	CRAG	Lentiviral vector	Neuroprotection	[52]
	NPY	AAV vectors	Neuroprotection	[53]

CYP46A1–cholesterol 24-hydroxylase; iRNA–Interference RNA; CRAG–collapsing response mediator protein (CRMP)-associated molecule (CRAM[CRMP-5])-associated GTPase; NPY–neuropeptide Y; MJD–Machado-Joseph Disease AAV–Adeno-associated virus; Tg–transgenic.

## 3. Gene Silencing Strategies

PolyQ SCAs have their underlying cause in single genetic factors, which influences a diverse set of downstream molecular pathways that contribute for disease progression [16]. Additionally, in vivo studies, regarding mutant *ATXN3* RNA-derived toxicity, have reported that the expression of untranslated transcripts with abnormally expanded CAG repeats lead to cell degeneration in *Drosophila*, *Caenorhabditis elegans* and mouse models [54,55,56]. This toxicity may be due to (i) the formation of expanded CAG RNAs foci, which can sequester proteins implicated in alternative splicing [57]; (ii) interference with nucleolar function [58] or (iii) silencing of the expression of certain genes [59]. The length of untranslated CAG transcripts was also shown to directly influence the toxicity of such RNA molecules, as the expression of transcripts with increased CAG length, deteriorated motor phenotype [55]. Considering these findings, silencing the expression of such pathological RNAs will result in a lack of toxic protein translation, while also eliminating the toxicity exerted by the RNA molecules themself. Therefore, the most straightforward therapeutic approach for PolyQ SCAs would be to silence the expression of the gene containing the disease-causing mutation. This may be achieved by promoting RNA degradation, skipping the mutant exon, impairing protein translation, correcting the pathological mutation or preventing gene translation altogether. Ultimately, such gene silencing strategies would allow to act at the earliest steps possible, admittedly preventing disease onset or progression [16].

### 3.1. RNAi-Based Gene Silencing Strategies

Endogenous RNA interference (RNAi) pathway is driven by microRNAs (miRNAs), endogenous non-coding RNAs comprised of approximately 22 nucleotides. These non-coding RNAs have been found to regulate different cellular processes, from cell proliferation, and development, to cell death [60]. Taking advantage of the discovery of this versatile tool for regulating target genes, researchers started to synthesize artificial RNAi molecules constituted by 21 to 23 nucleotides, with the aim of inhibiting genes of interest and observing the outcome, as part of fundamental research efforts. This opened the door for designing oligonucleotides capable of silencing a gene responsible for a particular pathology, enabling the treatment of dominant genetic diseases such as PolyQ SCAs [61].

Experimentally, the mechanism of RNAi can thus be triggered by different RNA molecules: miRNAs, siRNAs and shRNAs [62], which may be delivered in different ways. They can be introduced into the cell as small interfering RNAs (siRNAs), similarly to a protein-based therapy, or via plasmids and viral vectors, which incorporate into the genome and are endogenously expressed in the form of short hairpin RNAs (shRNAs), which, contrary to siRNAs, result in enduring gene silencing [63,64].

In the case of nucleus-bound RNA molecules such as miRNA and shRNA, the RNAi pathway starts with the expression of those RNAs transcripts, containing hairpin structures, and their processing by Drosha. The processed RNA molecules are then transported to the cytoplasm via exportin-5, a nuclear membrane protein. Once in the cytoplasm the RNAi pathway is common to the nuclear and cytoplasmic RNAs, such as siRNAs. Upon entering the cytoplasm, RNAi molecules are processed by Dicer, giving rise to a mature RNAi molecule. The antisense strand from the mature RNAi molecule is then loaded onto a protein complex named RNA-induced silencing complex (RISC), which will recognize a specific complementary mRNA. Upon biding, two silencing pathways can occur: (i) a RNAse-mediated degradation when the target sequence mRNA is 100% complementary to the RNAi molecule, which results in digestion of that target sequence, or (ii) repression of mRNA translation when complementarity is partial [62,65].

#### 3.1.1. Short Hairpin and Small Interfering RNAs Mediated Silencing

The current foundations for non-allele-specific silencing using artificial RNAi molecules in PolyQ SCAs were laid in 2004 by Xia and his colleagues (Table 4). In their study, delivery of AAV1 expressing shRNAs targeting *ATXN1* to the cerebellum of a SCA1 mouse model, knocked-down ataxin-1 levels significantly and led to motor improvements, cerebellar morphology rescue and reduction of ataxin-1 aggregates in Purkinje cells [66]. Following these findings, the same group improved the strategy’s safety and efficacy by cloning the previously used shRNA into a miRNA backbone, and by delivering them to the cerebellar nuclei providing a broader biodistribution [47,67,68]. Viral-mediated knockdown of endogenous *ATXN3* in a non-allele-specific manner also proved to be safe and efficient in SCA3/MJD models. Indeed, *ATXN3* knockdown resulted in a reduction of mutant *ATXN3* levels and decreased considerably the number of ataxin-3 inclusions as well as neuropathological features [48].

Mouse and *C. elegans ATXN3* knockout models are viable and display no abnormal phenotype [69,70]. However, the absence of Ataxin-3 affects many transduction pathways and alters the regulation of a large set of genes, which may result in deleterious effects [71]. Furthermore, the loss of WT Ataxin-3 during long periods of time has not been fully assessed. Taking this into account, a silencing strategy should be whenever possible targeted only to the mutant allele, allowing normal gene functions not to be disrupted. Although more technically challenging, allele-specific approaches allow RNAi molecules to distinguishing between wild-type and mutant alleles. In order to develop allele-specific strategies targeting mutant alleles, the presence of genetic variants such as single-nucleotide-polymorphisms (SNP), only common in the patient population must be first identified [72].

Among PolyQ SCAs, SCA3/MJD has seen the most extensive efforts made towards allele-specific silencing. Li and colleagues were the first to successfully target mutant *ATXN3* and reduce its levels in cell cultures, while WT levels were only slightly reduced [73]. Later, a study performed in a SCA3/MJD rat model established the proof-of-concept for allele-specific silencing in the disease [72]. This pioneer study in conjunction with a following study, demonstrated that viral-mediated silencing of mutant *ATXN3* was accompanied by a mitigation of neuropathological deficits [72,74]. Additionally, Nóbrega and colleagues also showed the recovery of neuropathological and motor features associated with SCA3/MJD after disease onset, following mutant *ATXN3* silencing in a transgenic mouse model [75]. Furthermore, the long-term expression of the shRNA used in these studies did not lead to toxic effects, in a recent safety assessment [64]. In an alternative approach, siRNAs targeting mutant *ATXN3* were encapsulated in SNALPs and delivered intravenously to different SCA3/MJD mouse models. This resulted in efficient selective silencing and improved motor behavior and neuropathology [76].

In vitro studies performed in SCA7 patient derived fibroblasts demonstrated that allele-specific siRNAs are able to selectively silence *ATXN7*. The siRNAs targeting a SNP within mutant *ATXN7* led to a knockdown of the mutant transcript and mitigation of disease-relevant phenotype [77], while a siRNA targeting the CAG repeat region in *ATXN7*, only resulted in decrease of mutant Ataxin-7 at a protein level [78].

Most recently, viral mediated delivery of the same shRNAs targeting the CAG repeat tract to SCA3/MJD and SCA7 patient derived fibroblasts, resulted in robust allele-specific silencing of mutant *ATXN3* and *ATXN7*, respectively [79].

**Table 4:** Selected siRNA and ShRNA based studies in polyglutamine spinocerebellar ataxias.

Disease	Target	Allele Specificity	Technology	Experimental Systems	Delivery	References
SCA1	Ataxin-1	Non-specific	shRNA	SCA1 transgenic mouse model	AAV-mediated transduction	[66]
SCA3/MJD	Mutant Ataxin-3	Allele-specific	siRNA	HEK 293T cells	Transfection	[73]
	Mutant Ataxin-3	Allele-specific	shRNA	LV-induced SCA3/MJD rat model	LV-mediated transduction	[72]
	Mutant Ataxin-3	Allele-specific	siRNA	SCA3/MJD transgenic mouse model	SNALPs-mediated transduction	[76]
	Mutant Ataxin-3	Allele-specific	shRNA	Patient derived fibroblasts	LV-mediated transduction	[79]
	Ataxin-3	Non-specific	shRNA	LV-induced SCA3/MJD rat model	LV-mediated transduction	[48]
SCA7	Mutant Ataxin-7	Allele-specific	siRNA	Patient derived fibroblasts	Transfection	[77]
	Mutant Ataxin-7	Allele-specific	siRNA	Patient derived fibroblasts	Transfection	[78]
	Mutant Ataxin-7	Allele-specific	shRNA	Patient derived fibroblasts	LV-mediated transduction	[79]

SNALPs–stable nucleic acid lipid particle; LV–lentiviral; AAV–Adeno-associated virus.

#### 3.1.2. MicroRNA-Mediated Silencing

Considering that miRNAs physiologically regulate gene expression, miRNA profiling studies in cells from PolyQ SCAs patients, often result in the identification of miRNAs responsible for regulating the disease-causing gene in a pathological context [80]. This allows to harness the natural silencing properties of miRNAs to knockdown mutant genes through artificial or mimic miRNA molecules. In fact, the expression of artificial miRNAs directed towards *ATXN1*, *ATXN3*, and *ATXN7* in cell cultures and/or mouse models and non-human primates have proven efficient in reducing mRNA and protein levels of all three genes (Table 5) [47,68,81,82,83,84,85,86].

For example, the administration of a miR-25 mimic to SCA3/MJD cellular models decreased ataxin-3 levels and aggregation, while also reducing apoptosis and therefore increasing cell viability [87]. In another study, miR-9, miR-181a and miR-494 were found to be downregulated and associated with neuropathology in SCA3/MJD. Upon reestablishment of these miRNAs, mutant ataxin-3 levels were reduced in both in vitro and in vivo models [88].

Following the identification of miR-3191 as a potential therapeutic miRNA for SCA6, this miRNA was delivered via viral vector to a SCA6 mouse model. This resulted in an amelioration of motor disease related features and reduced Purkinje cell degeneration [89]. A study in SCA7 knock-in mice and N2a cell cultures, revealed that the administration of a miR-124 mimic led to a decrease in both Ataxin-7 and lnc-SCA7 expression [90]. As for SCA1 cell models, ataxin-1 levels were found to be decreased upon overexpression of miR-19, miR-101 and miR-130 [91]. Additionally, another study also reported that miR-144 overexpression significantly decreased Ataxin-1 protein levels in HEK 293T cells [92].

The reports compiled in this section, contribute to strengthen RNAi-based strategies as a viable therapeutic approach for PolyQ SCAs, capable of efficiently promoting gene silencing whether through an allele-specific or non-allele-specific manner, although the former is still limited to SCA3/MJD and SCA7. In order to develop future allele-specific strategies for PolyQ SCAs, further knowledge in patient genetic variants is required.

**Table 5:** Selected microRNAs studies in polyglutamine spinocerebellar ataxias.

Disease	microRNAs	Target	Experimental System	Delivery	References
SCA1	Artificial miRNA	*ATXN1*	C2C12 cells, SCA1 transgenic mouse model and non-human primates	AVV-mediated transduction	[67,68,81,83]
	miR-19, miR-101 and miR-130 mimic	*ATXN1*	HEK 293T, HeLa and MCF7 cells	Transfection	[91]
	miR-144 mimic	*ATXN1*	HEK 293T cells	Transfection	[92]
SCA3/MJD	Artificial miRNA	*ATXN3*	SCA3/MJD transgenic mouse model	AAV-mediated transduction	[82,86]
	miR-25 mimic	*ATXN3*	HEK 293T and SH-S5Y5 cells	Transfection	[87]
	miR-9, miR-181a and miR-494 mimics	*ATXN3*	HEK 293T cells and LV-induced SCA3/MJD mouse model	LV-mediated transduction	[88]
SCA6	miR-3191-5p	*CACNA1A*	AAV-induced SCA6 mouse model	AAV-mediated transduction	[89]
SCA7	Artificial miRNA	*ATXN7*	SCA7 transgenic mouse model	AAV-mediated transduction	[84,85]
	miR-124 mimic	Lnc-SCA7 and ataxin-7	N2a cells	Transfection	[90]

Lnc–Long non-coding; LV–lentiviral; AAV–Adeno-associated virus; SCA–Spinocerebellar ataxia; MJD–Machado-Joseph Disease; *ATXN*–Ataxin gene; *CACNA1A*–Calcium voltage-gated channel subunit α1 G.

### 3.2. Antisense Oligonucleotides

Antisense oligonucleotides (ASOs) are synthetic single-stranded DNA molecules, capable of hybridizing to target mRNA molecules through Watson-Crick base pairing and alter their functions, and thus allowing to artificially modulate gene expression via different mechanisms [93]. These mechanisms may be divided in two groups: RNAse H-dependent mRNA degradation and RNAse H-independent. In order to mediate RNAse H-dependent mRNA degradation, a portion of nucleotides in the 2′ position of the ASOs molecules must remain unmodified [94,95]. Following the formation of the mRNA:DNA(ASO) duplex and its recognition by RNAse H, the target mRNA molecule is cleaved, while the artificial oligonucleotide molecule remains intact [95]. In the case of RNAse H-independent RNA modulation, completely 2′-modified ASOs may be used to mediate several processes where mRNA degradation is not the outcome. This mechanism can be employed to modulate mRNA splicing events, which in the context of PolyQ SCAs could enable skipping the exons containing the pathological mutation [96,97].

ASOs have been employed in several PolyQ SCAs studies as a potential silencing strategy (Table 6). In 2013, Evers and colleagues demonstrated that ASOs were able to mediate exon skipping of the *ATXN3* exon containing the CAG repeats in cell and mouse models, without any apparent deleterious effects [96]. Additionally, the same group also showed that this exon skipping strategy in a transgenic SCA3/MJD mouse model led to a reduction in ataxin-3 insolubility and nuclear accumulation of the protein [98]. A study employing ASOs to promote ataxin-3 RNA degradation resulted in a reduction of mutant ataxin-3 protein levels in cell and mouse models [99]. Following this previous study, the most promising ASO was chosen and delivered to a SCA3/MJD mouse model. Upon ASO delivery, mitigation of several disease-associated phenotypes was observed [100].

Regarding other PolyQ SCAs, recent studies applying ASOs mediated RNA suppression to reduce target gene expression and ameliorate disease phenotypes in mouse models have yielded promising results. In fact, studies in SCA1 and SCA2 mouse models, revealed that upon intracerebroventricular (IVC) injection, expression of *ATXN1* and *ATXN2* mRNA levels were significantly decrease and motor deficits mitigated [101,102]. Noteworthy, ASO-mediated *ATXN7* knockdown, in the eye of SCA7 mice, resulted in a considerable reduction of Ataxin-7 expression and protein aggregation, as well as the amelioration of several visual impairments [103].

More recently, a study performed in SCA3/MJD and SCA1 mouse models employed an ASO molecule, entitled (CUG)7, which had been previously developed to target the CAG stretches in Huntingtin [104]. In this study, researchers were able to successfully reduce the protein levels of mutant Ataxin-3 in SCA3/MJD patient derived fibroblasts and mouse model. As for SCA1, (CUG)7, was also able to reduce the protein levels on mutant ataxin-1 in both cell and mouse models [105].

In recent years ASOs-based approaches have seen a rise in interest. Promising results in several PolyQ SCAs established ASOs as a viable alternative to RNAi-based strategies. Notably, ASOs have greatly expanded their range in target diseases and improved upon existing ones in the last few years. Exon skipping mediated by ASOs may also become instrumental in the future potentially resolving mutant RNA derived toxicity, which has been identified as a possible pathological mechanism in PolyQ SCAs. Finally, multi-PolyQ disease approaches using ASOs such as the one developed by Kourkouta and colleagues [105] may mark the rise of ASOs as the future gold standard in oligonucleotide-mediated gene silencing.

**Table 6:** Selected ASOs-based studies in polyglutamine spinocerebellar ataxias.

Disease	Target	Mechanism	Delivery Method	References
SCA1	Ataxin-1	RNAse-H dependent degradation	ICV	[101]
	Ataxin-1	Translation hindrance	ICV	[105]
SCA2	Ataxin-2	RNAse-H dependent degradation	ICV	[102]
SCA3/MJD	Ataxin-3	Exon 9 and 10-skipping	ICV	[96]
	Ataxin-3	RNAse-H dependent degradation	ICV	[99]
	Ataxin-3	Exon10-skiping	ICV	[98]
	Ataxin-3	Translation hindrance and Exon10-skiping	ICV	[105]
SCA7	Ataxin-7	RNAse-H dependent degradation	IVI	[103]

IVI-intravitreal injection, IVC-intracerebroventricular injection; SCA—Spinocerebellar ataxia; MJD—Machado-Joseph Disease.

## 4. Gene Edition

The ideal therapeutic approach for PolyQ SCAs would focus on correcting the disease-causing mutation or permanently hinder expression of the mutant gene. Gene editing methods allow for precise and targetable modification of genome sequences, enabling the knockout of endogenous genes or the correction of existing defects, such as pathological CAG expansions [106,107,108,109]. Additionally, gene editing has been shown to efficiently induce modifications in a diverse set of cell types, in which neurons are included [110].

Endogenous DNA repair mechanisms can be harnessed to perform deliberate alteration in the target genome [111]. Typically, there are two major DNA repair pathways responsible for repairing double-stranded DNA breaks (DSB): nonhomologous end-joining (NHEJ), which occurs in the absence of template DNA molecules, and homology-directed repair (HDR), which occurs when they are present [112]. Targeted gene editing relying on these DNA repair mechanisms presupposes the ability to produce DSBs at particular genomic sites, i.e., at the vicinity of the sites intended to be altered. DSBs can be artificially introduced into the genome through the action of specific endonucleases, which can be engineered and repurposed for different applications [106]. Currently, endonucleases used in gene editing are grouped into four main classes: Meganucleases, Zinc finger Nucleases, TALENs and CRISPR/Cas systems [113,114,115,116]. The current gold standard CRISPR/Cas system consists of two distinct components–an endonuclease, usually the Cas9 protein from *Streptococcus pyogenes*, and a guide RNA molecule, that combines with Cas9 to form a ribonucleoprotein complex. The guide RNA directs the complex to its DNA target by binding to the particular nucleotide sequence through classic Watson and Crick base-pairing [116].

To date, the CRISPR/Cas9-mediated deletion of the expanded CAG repeat of *ATXN3* performed by Ouyang and colleagues represents the only reported gene editing study in PolyQ SCAs. In this study, induced pluripotent stem cells (iPSCs) derived from SCA3/MJD patients (SCA3-iPSCs) were transfected with two sgRNAs targeting sequences flanking the polyQ-encoding region. Results showed that the exon 10 containing the CAG repeats was deleted, while exon 9 and exon 11 remained unchanged. Furthermore, following gene editing, the ubiquitin-binding capacity of Ataxin-3 was maintained. Importantly, corrected SCA3-iPSCs were able to differentiate to both neural stem cells and neuronal cells, implying pluripotency was retained after treatment [109].

Although, this study was the first of its kind in PolyQ SCAs, studies performed on patient derived fibroblasts and a transgenic mouse model of Huntington’s disease (HD), a PolyQ disorder also arising from abnormal CAG repeat expansions, have also reported the successful removal of the CAG repeat tract though CRISPR/Cas9-mediated excision [117,118]. Nevertheless, this SCA3/MJD study established the proof-of-concept for PolyQ SCAs, demonstrating the feasibility of this strategy in this group of disorders. Hopefully in the near future, more research groups will investigate and develop this type of therapeutic strategies. Additionally, with future identification of additional disease associated SNPs, it will be arguably possible to direct the CRISPR/Cas9 system solely to the mutant allele. This would leave WT alleles unarmed and completely functional, while correcting the disease-causing mutation in the mutant allele.

## 5. Concluding Remarks and Future Perspectives

Considering the several traits shared between PolyQ SCAs, the potential therapeutic approaches here highlighted for one specific disorder may prove to be transversal for another. Several gene therapy strategies aimed at PolyQ SCAs have been used in pre-clinical models such as patient-derived cells, rodent models, and non-human primates. In the animal models, gene therapy administration demonstrated substantial improvements of disease-associated phenotypes and neuropathology, as well as reduction of the disease associated toxicity. Although no gene therapy-based approach has this far reached clinical trials, and considering the promising results reviewed in this article, such milestone may be achieved in the near future. Importantly, the most promising results obtained, were achieved when the therapeutic approach was directed to pathogenesis-related events, the disease-causing gene or its resulting transcripts. The aim of such approaches is either to counteract impairments in the autophagy pathway, promote neuroprotection, silence CAG-expanded transcripts or ultimately correct the pathological mutation.

PolyQ SCAs pathophysiology is not limited to a single pathway, which often complicates the application of therapeutic strategies aiming to act upon a single pathway or downstream protein interactions. Ideally the treatment for PolyQ SCAs would involve the correction of the mutation behind the disorder. An approach entailing this has been developed, however more gene editing approaches in this group of disorders need to be developed. Additionally, this type of therapy still faces numerous challenges in the future, such as long-term toxicity and safety risks. Until these risks are tackled, targeting mutant RNA transcripts represents the next best alternative for engaging at the most upstream point of the pathological cascade. Considering this, ASOs and RNAi-based strategies represent the most promising approaches in the near future, until gene editing takes the front stage. This line of thought is further reinforced by the advances observed in HD clinical trials. To date 4 studies employing ASOs (NCT02519036; NCT03342053; NCT03225833; NCT03225846) and 1 study employing miRNA (NCT04120493) have reached clinical trials, reflecting the potential silencing strategies have in the field of PolyQ disorders, namely PolyQ SCAs.

## Figures and Tables

**Table 1:** Classification of autosomal dominant spinocerebellar ataxia with *CAG expansions*.

PolyQ	Gene	Locus	Protein	Molecular Function	Repeats		
					Normal	Intermediate	Disease
SCA1	*ATXN1*	6p22.3	Ataxin-1	Transcription Factor Interactor	9–39	40	41–83
SCA2	*ATXN2*	12q24.12	Ataxin-2	RNA metabolism	<31	31–33	34–200
SCA3/MJD	*ATXN3*	14q32.12	Ataxin-3	Deubiquitinase	12–44	45–55	56–86
SCA6	*CACNA1A*	19p13.13	Calcium voltage-gated channel subunit alpha 1 G	Channel and Transcription Factor	<18	19	20–33
SCA7	*ATXN7*	3p14.1	Ataxin-7	Transcription Factor (SAGA Complex)	4–19	28–33	34–460
SCA17	*TBP*	6q27	TATA box-binding protein	Transcription Factor	25–40	-	41–66

SCA–Spinocerebellar ataxia; MJD–Machado-Joseph Disease; *ATXN*–Ataxin gene; *CACNA1A*–Calcium voltage-gated channel subunit α1 G; *TBP*–TATA box-binding protein; p–chromosome shorter arm; q–chromosome longer arm; SAGA complex–SPT-ADA-GCN5 acetyltransferase [1,2,3].

**Table 2:** PolyQ SCAs pathophysiological features and proposed therapeutic targets.

Pathophysiological Features	PolyQ SCAs Therapeutic Targets
Mutant RNA and protein derived toxicity	Disease-causing gene, mutant RNA
Impairments in protein degradation and aggregate clearance	Autophagy and ubiquitin-protease system, mutant protein aggregation
Formation of toxic protein fragments	Mutant protein cleavage and proteolytic enzymes
Deficient neuronal survival:	
Disfuncional cellular structures	Endoplasmic reticulum stress, mitochondrial functioning
Failure of cellular processes	Neuroprotective pathways, inflammation, excitotoxicity, synaptic signaling, calcium homeostasis, post transcription modifications, translation

## References

[1] Klockgether T., Mariotti C., Paulson H.L. (2019). Spinocerebellar ataxia. Nat. Rev. Dis. Primers.

[2] Artero Castro A., Machuca C., Rodriguez Jimenez F.J., Jendelova P., Erceg S. (2019). Short Review: Investigating ARSACS: Models for understanding cerebellar degeneration. Neuropathol. Appl. Neurobiol..

[3] Ceylan A.C., Acar Arslan E., Erdem H.B., Kavus H., Arslan M., Topaloglu H. (2020). Autosomal recessive spinocerebellar ataxia 18 caused by homozygous exon 14 duplication in GRID2 and review of the literature. Acta Neurol. Belg..

[4] Zanni G., Bertini E. (2018). X-linked ataxias. Handb. Clin. Neurol..

[5] Genis D., Ortega-Cubero S., San Nicolás H., Corral J., Gardenyes J., de Jorge L., López E., Campos B., Lorenzo E., Tonda R. (2018). Heterozygous *STUB1* mutation causes familial ataxia with cognitive affective syndrome (SCA48). J. Neurol..

[6] Ashizawa T., Oz G., Paulson H.L. (2018). Spinocerebellar ataxias: Prospects and challenges for therapy development. Nat. Rev. Neurol..

[7] Ruano L., Melo C., Silva M.C., Coutinho P. (2014). The global epidemiology of hereditary ataxia and spastic paraplegia: A systematic review of prevalence studies. Neuroepidemiology.

[8] Coutinho P., Ruano L., Loureiro J.L., Cruz V.T., Barros J., Tuna A., Barbot C., Guimaraes J., Alonso I., Silveira I. (2013). Hereditary ataxia and spastic paraplegia in Portugal: A population-based prevalence study. JAMA Neurol..

[9] Erichsen A.K., Koht J., Stray-Pedersen A., Abdelnoor M., Tallaksen C.M. (2009). Prevalence of hereditary ataxia and spastic paraplegia in southeast Norway: A population-based study. Brain.

[10] Tsuji S., Onodera O., Goto J., Nishizawa M., Study Group on Ataxic D. (2008). Sporadic ataxias in Japan--a population-based epidemiological study. Cerebellum.

[11] Monin M.L., Tezenas du Montcel S., Marelli C., Cazeneuve C., Charles P., Tallaksen C., Forlani S., Stevanin G., Brice A., Durr A. (2015). Survival and severity in dominant cerebellar ataxias. Ann. Clin. Transl. Neurol..

[12] La Spada A.R. (1997). Trinucleotide repeat instability: Genetic features and molecular mechanisms. Brain Pathol..

[13] Buijsen R.A.M., Toonen L.J.A., Gardiner S.L., van Roon-Mom W.M.C. (2019). Genetics, Mechanisms, and Therapeutic Progress in Polyglutamine Spinocerebellar Ataxias. Neurotherapeutics.

[14] Paulson H.L., Shakkottai V.G., Clark H.B., Orr H.T. (2017). Polyglutamine spinocerebellar ataxias—from genes to potential treatments. Nat. Rev. Neurosci..

[15] Coarelli G., Brice A., Durr A. (2018). Recent advances in understanding dominant spinocerebellar ataxias from clinical and genetic points of view. F1000Res.

[16] Bauer P.O., Nukina N. (2009). The pathogenic mechanisms of polyglutamine diseases and current therapeutic strategies. J. Neurochem..

[17] Evers M.M., Toonen L.J., van Roon-Mom W.M. (2014). Ataxin-3 protein and RNA toxicity in spinocerebellar ataxia type 3: Current insights and emerging therapeutic strategies. Mol. Neurobiol..

[18] Jimenez-Sanchez M., Thomson F., Zavodszky E., Rubinsztein D.C. (2012). Autophagy and polyglutamine diseases. Prog. Neurobiol..

[19] Durr A. (2010). Autosomal dominant cerebellar ataxias: Polyglutamine expansions and beyond. Lancet Neurol..

[20] Matilla-Duenas A., Ashizawa T., Brice A., Magri S., McFarland K.N., Pandolfo M., Pulst S.M., Riess O., Rubinsztein D.C., Schmidt J. (2014). Consensus paper: Pathological mechanisms underlying neurodegeneration in spinocerebellar ataxias. Cerebellum.

[21] Matos C.A., de Almeida L.P., Nobrega C. (2019). Machado-Joseph disease/spinocerebellar ataxia type 3: Lessons from disease pathogenesis and clues into therapy. J. Neurochem..

[22] Katsuno M., Watanabe H., Yamamoto M., Sobue G. (2014). Potential therapeutic targets in polyglutamine-mediated diseases. Expert Rev. Neurother..

[23] Sullivan R., Yau W.Y., O’Connor E., Houlden H. (2019). Spinocerebellar ataxia: An update. J. Neurol..

[24] Marcelo A., Brito F., Carmo-Silva S., Matos C.A., Alves-Cruzeiro J., Vasconcelos-Ferreira A., Koppenol R., Mendonca L., de Almeida L.P., Nobrega C. (2019). Cordycepin activates autophagy through AMPK phosphorylation to reduce abnormalities in Machado-Joseph disease models. Hum. Mol. Genet..

[25] Santana M.M., Paixao S., Cunha-Santos J., Silva T.P., Trevino-Garcia A., Gaspar L.S., Nobrega C., Nobre R.J., Cavadas C., Greif H. (2020). Trehalose alleviates the phenotype of Machado-Joseph disease mouse models. J. Transl. Med..

[26] Mendonca L.S., Nobrega C., Tavino S., Brinkhaus M., Matos C., Tome S., Moreira R., Henriques D., Kaspar B.K., Pereira de Almeida L. (2019). Ibuprofen enhances synaptic function and neural progenitors proliferation markers and improves neuropathology and motor coordination in Machado-Joseph disease models. Hum. Mol. Genet..

[27] Nakamura K., Mieda T., Suto N., Matsuura S., Hirai H. (2015). Mesenchymal stem cells as a potential therapeutic tool for spinocerebellar ataxia. Cerebellum.

[28] Oliveira Miranda C., Marcelo A., Silva T.P., Barata J., Vasconcelos-Ferreira A., Pereira D., Nóbrega C., Duarte S., Barros I., Alves J. (2018). Repeated Mesenchymal Stromal Cell Treatment Sustainably Alleviates Machado-Joseph Disease. Mol. Ther..

[29] Cendelin J. (2016). Transplantation and Stem Cell Therapy for Cerebellar Degenerations. Cerebellum.

[30] Chintawar S., Hourez R., Ravella A., Gall D., Orduz D., Rai M., Bishop D.P., Geuna S., Schiffmann S.N., Pandolfo M. (2009). Grafting neural precursor cells promotes functional recovery in an SCA1 mouse model. J. Neurosci..

[31] Mendonca L.S., Nobrega C., Hirai H., Kaspar B.K., Pereira de Almeida L. (2015). Transplantation of cerebellar neural stem cells improves motor coordination and neuropathology in Machado-Joseph disease mice. Brain.

[32] Naldini L. (2015). Gene therapy returns to centre stage. Nature.

[33] Kaufmann K.B., Buning H., Galy A., Schambach A., Grez M. (2013). Gene therapy on the move. EMBO Mol. Med..

[34] Nóbrega C., Mendonça L., Matos C.A. (2020). Gene Therapy Strategies: Gene Augmentation. A Handbook of Gene and Cell Therapy.

[35] Gatchel J.R., Watase K., Thaller C., Carson J.P., Jafar-Nejad P., Shaw C., Zu T., Orr H.T., Zoghbi H.Y. (2008). The insulin-like growth factor pathway is altered in spinocerebellar ataxia type 1 and type 7. Proc. Natl. Acad. Sci. USA.

[36] Zarouchlioti C., Parfitt D.A., Li W., Gittings L.M., Cheetham M.E. (2018). DNAJ Proteins in neurodegeneration: Essential and protective factors. Philos. Trans. R. Soc. Lond. B Biol. Sci..

[37] Cortes C.J., La Spada A.R. (2015). Autophagy in polyglutamine disease: Imposing order on disorder or contributing to the chaos?. Mol. Cell Neurosci..

[38] Ravikumar B., Sarkar S., Davies J.E., Futter M., Garcia-Arencibia M., Green-Thompson Z.W., Jimenez-Sanchez M., Korolchuk V.I., Lichtenberg M., Luo S. (2010). Regulation of mammalian autophagy in physiology and pathophysiology. Physiol. Rev..

[39] Hara T., Nakamura K., Matsui M., Yamamoto A., Nakahara Y., Suzuki-Migishima R., Yokoyama M., Mishima K., Saito I., Okano H. (2006). Suppression of basal autophagy in neural cells causes neurodegenerative disease in mice. Nature.

[40] Komatsu M., Waguri S., Chiba T., Murata S., Iwata J., Tanida I., Ueno T., Koike M., Uchiyama Y., Kominami E. (2006). Loss of autophagy in the central nervous system causes neurodegeneration in mice. Nature.

[41] Lee L.C., Chen C.M., Wang P.R., Su M.T., Lee-Chen G.J., Chang C.Y. (2014). Role of high mobility group box 1 (HMGB1) in SCA17 pathogenesis. PLoS ONE.

[42] Nobrega C., Mendonca L., Marcelo A., Lamaziere A., Tome S., Despres G., Matos C.A., Mechmet F., Langui D., den Dunnen W. (2019). Restoring brain cholesterol turnover improves autophagy and has therapeutic potential in mouse models of spinocerebellar ataxia. Acta Neuropathol..

[43] Nascimento-Ferreira I., Santos-Ferreira T., Sousa-Ferreira L., Auregan G., Onofre I., Alves S., Dufour N., Colomer Gould V.F., Koeppen A., Déglon N. (2011). Overexpression of the autophagic beclin-1 protein clears mutant ataxin-3 and alleviates Machado-Joseph disease. Brain J. Neurol..

[44] Nascimento-Ferreira I., Nóbrega C., Vasconcelos-Ferreira A., Onofre I., Albuquerque D., Aveleira C., Hirai H., Déglon N., Pereira de Almeida L. (2013). Beclin 1 mitigates motor and neuropathological deficits in genetic mouse models of Machado-Joseph disease. Brain J. Neurol..

[45] Ruegsegger C., Stucki D.M., Steiner S., Angliker N., Radecke J., Keller E., Zuber B., Ruegg M.A., Saxena S. (2016). Impaired mTORC1-Dependent Expression of Homer-3 Influences SCA1 Pathophysiology. Neuron.

[46] Silva A., de Almeida A.V., Macedo-Ribeiro S. (2018). Polyglutamine expansion diseases: More than simple repeats. J. Struct. Biol..

[47] Keiser M.S., Geoghegan J.C., Boudreau R.L., Lennox K.A., Davidson B.L. (2013). RNAi or overexpression: Alternative therapies for Spinocerebellar Ataxia Type 1. Neurobiol. Dis..

[48] Alves S., Nascimento-Ferreira I., Dufour N., Hassig R., Auregan G., Nóbrega C., Brouillet E., Hantraye P., Pedroso de Lima M.C., Déglon N. (2010). Silencing ataxin-3 mitigates degeneration in a rat model of Machado-Joseph disease: No role for wild-type ataxin-3?. Hum. Mol. Genet..

[49] Nobrega C., Carmo-Silva S., Albuquerque D., Vasconcelos-Ferreira A., Vijayakumar U.G., Mendonca L., Hirai H., de Almeida L.P. (2015). Re-establishing ataxin-2 downregulates translation of mutant ataxin-3 and alleviates Machado-Joseph disease. Brain.

[50] Matos C.A., Almeida L.P., Nobrega C. (2017). Proteolytic Cleavage of Polyglutamine Disease-Causing Proteins: Revisiting the Toxic Fragment Hypothesis. Curr. Pharm. Des..

[51] Simões A.T., Gonçalves N., Koeppen A., Déglon N., Kügler S., Duarte C.B., Pereira de Almeida L. (2012). Calpastatin-mediated inhibition of calpains in the mouse brain prevents mutant ataxin 3 proteolysis, nuclear localization and aggregation, relieving Machado-Joseph disease. Brain J. Neurol..

[52] Torashima T., Koyama C., Iizuka A., Mitsumura K., Takayama K., Yanagi S., Oue M., Yamaguchi H., Hirai H. (2008). Lentivector-mediated rescue from cerebellar ataxia in a mouse model of spinocerebellar ataxia. EMBO Rep..

[53] Duarte-Neves J., Gonçalves N., Cunha-Santos J., Simões A.T., den Dunnen W.F., Hirai H., Kügler S., Cavadas C., Pereira de Almeida L. (2015). Neuropeptide Y mitigates neuropathology and motor deficits in mouse models of Machado-Joseph disease. Hum. Mol. Genet..

[54] Li L.B., Yu Z., Teng X., Bonini N.M. (2008). RNA toxicity is a component of ataxin-3 degeneration in Drosophila. Nature.

[55] Wang L.C., Chen K.Y., Pan H., Wu C.C., Chen P.H., Liao Y.T., Li C., Huang M.L., Hsiao K.M. (2011). Muscleblind participates in RNA toxicity of expanded CAG and CUG repeats in Caenorhabditis elegans. Cell. Mol. Life Sci..

[56] Hsu R.J., Hsiao K.M., Lin M.J., Li C.Y., Wang L.C., Chen L.K., Pan H. (2011). Long tract of untranslated CAG repeats is deleterious in transgenic mice. PLoS ONE.

[57] Mykowska A., Sobczak K., Wojciechowska M., Kozlowski P., Krzyzosiak W.J. (2011). CAG repeats mimic CUG repeats in the misregulation of alternative splicing. Nucleic Acids Res..

[58] Tsoi H., Lau T.C., Tsang S.Y., Lau K.F., Chan H.Y. (2012). CAG expansion induces nucleolar stress in polyglutamine diseases. Proc. Natl. Acad. Sci. USA.

[59] Krol J., Fiszer A., Mykowska A., Sobczak K., de Mezer M., Krzyzosiak W.J. (2007). Ribonuclease dicer cleaves triplet repeat hairpins into shorter repeats that silence specific targets. Mol. Cell.

[60] Bartel D.P. (2004). MicroRNAs. Cell.

[61] Setten R.L., Rossi J.J., Han S.P. (2019). The current state and future directions of RNAi-based therapeutics. Nat. Rev. Drug Discov..

[62] Matos C.A., Carmona V., Vijayakumar U.G., Lopes S., Albuquerque P., Conceição M., Nobre R.J., Nóbrega C., de Almeida L.P. (2018). Gene Therapies for Polyglutamine Diseases. Adv. Exp. Med. Biol..

[63] Han H. (2018). RNA Interference to Knock Down Gene Expression. Methods Mol. Biol..

[64] Nóbrega C., Codêsso J.M., Mendonça L., Pereira de Almeida L. (2019). RNA Interference Therapy for Machado-Joseph Disease: Long-Term Safety Profile of Lentiviral Vectors Encoding Short Hairpin RNAs Targeting Mutant Ataxin-3. Hum. Gene Ther..

[65] Bobbin M.L., Rossi J.J. (2016). RNA Interference (RNAi)-Based Therapeutics: Delivering on the Promise?. Annu. Rev. Pharm. Toxicol..

[66] Xia H., Mao Q., Eliason S.L., Harper S.Q., Martins I.H., Orr H.T., Paulson H.L., Yang L., Kotin R.M., Davidson B.L. (2004). RNAi suppresses polyglutamine-induced neurodegeneration in a model of spinocerebellar ataxia. Nat. Med..

[67] Keiser M.S., Boudreau R.L., Davidson B.L. (2014). Broad therapeutic benefit after RNAi expression vector delivery to deep cerebellar nuclei: Implications for spinocerebellar ataxia type 1 therapy. Mol. Ther. J. Am. Soc. Gene Ther..

[68] Keiser M.S., Kordower J.H., Gonzalez-Alegre P., Davidson B.L. (2015). Broad distribution of ataxin 1 silencing in rhesus cerebella for spinocerebellar ataxia type 1 therapy. Brain J. Neurol..

[69] Rodrigues A.J., Coppola G., Santos C., Costa Mdo C., Ailion M., Sequeiros J., Geschwind D.H., Maciel P. (2007). Functional genomics and biochemical characterization of the C. elegans orthologue of the Machado-Joseph disease protein ataxin-3. FASEB J. Off. Publ. Fed. Am. Soc. Exp. Biol..

[70] Schmitt I., Linden M., Khazneh H., Evert B.O., Breuer P., Klockgether T., Wuellner U. (2007). Inactivation of the mouse Atxn3 (ataxin-3) gene increases protein ubiquitination. Biochem. Biophys. Res. Commun..

[71] Zeng L., Zhang D., McLoughlin H.S., Zalon A.J., Aravind L., Paulson H.L. (2018). Loss of the Spinocerebellar Ataxia type 3 disease protein ATXN3 alters transcription of multiple signal transduction pathways. PLoS ONE.

[72] Alves S., Nascimento-Ferreira I., Auregan G., Hassig R., Dufour N., Brouillet E., Pedroso de Lima M.C., Hantraye P., Pereira de Almeida L., Déglon N. (2008). Allele-specific RNA silencing of mutant ataxin-3 mediates neuroprotection in a rat model of Machado-Joseph disease. PLoS ONE.

[73] Li Y., Yokota T., Matsumura R., Taira K., Mizusawa H. (2004). Sequence-dependent and independent inhibition specific for mutant ataxin-3 by small interfering RNA. Ann. Neurol..

[74] Nóbrega C., Nascimento-Ferreira I., Onofre I., Albuquerque D., Déglon N., de Almeida L.P. (2014). RNA interference mitigates motor and neuropathological deficits in a cerebellar mouse model of Machado-Joseph disease. PLoS ONE.

[75] Nóbrega C., Nascimento-Ferreira I., Onofre I., Albuquerque D., Hirai H., Déglon N., de Almeida L.P. (2013). Silencing mutant ataxin-3 rescues motor deficits and neuropathology in Machado-Joseph disease transgenic mice. PLoS ONE.

[76] Conceição M., Mendonça L., Nóbrega C., Gomes C., Costa P., Hirai H., Moreira J.N., Lima M.C., Manjunath N., Pereira de Almeida L. (2016). Intravenous administration of brain-targeted stable nucleic acid lipid particles alleviates Machado-Joseph disease neurological phenotype. Biomaterials.

[77] Scholefield J., Watson L., Smith D., Greenberg J., Wood M.J. (2014). Allele-specific silencing of mutant Ataxin-7 in SCA7 patient-derived fibroblasts. Eur. J. Hum. Genet..

[78] Fiszer A., Wroblewska J.P., Nowak B.M., Krzyzosiak W.J. (2016). Mutant CAG Repeats Effectively Targeted by RNA Interference in SCA7 Cells. Genes.

[79] Kotowska-Zimmer A., Ostrovska Y., Olejniczak M. (2020). Universal RNAi Triggers for the Specific Inhibition of Mutant Huntingtin, Atrophin-1, Ataxin-3, and Ataxin-7 Expression. Mol. Ther. Nucleic Acids.

[80] Krauss S., Nalavade R., Weber S., Carter K., Evert B.O. (2019). Upregulation of miR-25 and miR-181 Family Members Correlates with Reduced Expression of ATXN3 in Lymphocytes from SCA3 Patients. Microrna (ShariqahUnited Arab Emir.).

[81] Boudreau R.L., Martins I., Davidson B.L. (2009). Artificial microRNAs as siRNA shuttles: Improved safety as compared to shRNAs in vitro and in vivo. Mol. Ther. J. Am. Soc. Gene Ther..

[82] Costa Mdo C., Luna-Cancalon K., Fischer S., Ashraf N.S., Ouyang M., Dharia R.M., Martin-Fishman L., Yang Y., Shakkottai V.G., Davidson B.L. (2013). Toward RNAi therapy for the polyglutamine disease Machado-Joseph disease. Mol. Ther. J. Am. Soc. Gene Ther..

[83] Keiser M.S., Kordasiewicz H.B., McBride J.L. (2016). Gene suppression strategies for dominantly inherited neurodegenerative diseases: Lessons from Huntington’s disease and spinocerebellar ataxia. Hum. Mol. Genet..

[84] Ramachandran P.S., Bhattarai S., Singh P., Boudreau R.L., Thompson S., Laspada A.R., Drack A.V., Davidson B.L. (2014). RNA interference-based therapy for spinocerebellar ataxia type 7 retinal degeneration. PLoS ONE.

[85] Ramachandran P.S., Boudreau R.L., Schaefer K.A., La Spada A.R., Davidson B.L. (2014). Nonallele specific silencing of ataxin-7 improves disease phenotypes in a mouse model of SCA7. Mol. Ther. J. Am. Soc. Gene Ther..

[86] Rodríguez-Lebrón E., Costa Mdo C., Luna-Cancalon K., Peron T.M., Fischer S., Boudreau R.L., Davidson B.L., Paulson H.L. (2013). Silencing mutant ATXN3 expression resolves molecular phenotypes in SCA3 transgenic mice. Mol. Ther. J. Am. Soc. Gene Ther..

[87] Huang F., Zhang L., Long Z., Chen Z., Hou X., Wang C., Peng H., Wang J., Li J., Duan R. (2014). miR-25 alleviates polyQ-mediated cytotoxicity by silencing ATXN3. FEBS Lett..

[88] Carmona V., Cunha-Santos J., Onofre I., Simões A.T., Vijayakumar U., Davidson B.L., Pereira de Almeida L. (2017). Unravelling Endogenous MicroRNA System Dysfunction as a New Pathophysiological Mechanism in Machado-Joseph Disease. Mol. Ther. J. Am. Soc. Gene Ther..

[89] Miyazaki Y., Du X., Muramatsu S., Gomez C.M. (2016). An miRNA-mediated therapy for SCA6 blocks IRES-driven translation of the CACNA1A second cistron. Sci. Transl. Med..

[90] Tan J.Y., Vance K.W., Varela M.A., Sirey T., Watson L.M., Curtis H.J., Marinello M., Alves S., Steinkraus B., Cooper S. (2014). Cross-talking noncoding RNAs contribute to cell-specific neurodegeneration in SCA7. Nat. Struct. Mol. Biol..

[91] Lee Y., Samaco R.C., Gatchel J.R., Thaller C., Orr H.T., Zoghbi H.Y. (2008). miR-19, miR-101 and miR-130 co-regulate ATXN1 levels to potentially modulate SCA1 pathogenesis. Nat. Neurosci..

[92] Persengiev S., Kondova I., Otting N., Koeppen A.H., Bontrop R.E. (2011). Genome-wide analysis of miRNA expression reveals a potential role for miR-144 in brain aging and spinocerebellar ataxia pathogenesis. Neurobiol. Aging.

[93] Zamecnik P.C., Stephenson M.L. (1978). Inhibition of Rous sarcoma virus replication and cell transformation by a specific oligodeoxynucleotide. Proc. Natl. Acad. Sci. USA.

[94] Monia B.P., Lesnik E.A., Gonzalez C., Lima W.F., McGee D., Guinosso C.J., Kawasaki A.M., Cook P.D., Freier S.M. (1993). Evaluation of 2’-modified oligonucleotides containing 2’-deoxy gaps as antisense inhibitors of gene expression. J. Biol. Chem..

[95] Walder R.Y., Walder J.A. (1988). Role of RNase H in hybrid-arrested translation by antisense oligonucleotides. Proc. Natl. Acad. Sci. USA.

[96] Evers M.M., Tran H.D., Zalachoras I., Pepers B.A., Meijer O.C., den Dunnen J.T., van Ommen G.J., Aartsma-Rus A., van Roon-Mom W.M. (2013). Ataxin-3 protein modification as a treatment strategy for spinocerebellar ataxia type 3: Removal of the CAG containing exon. Neurobiol. Dis..

[97] Sazani P., Kole R. (2003). Therapeutic potential of antisense oligonucleotides as modulators of alternative splicing. J. Clin. Investig..

[98] Toonen L.J.A., Rigo F., van Attikum H., van Roon-Mom W.M.C. (2017). Antisense Oligonucleotide-Mediated Removal of the Polyglutamine Repeat in Spinocerebellar Ataxia Type 3 Mice. Mol. Ther. Nucleic Acids.

[99] Moore L.R., Rajpal G., Dillingham I.T., Qutob M., Blumenstein K.G., Gattis D., Hung G., Kordasiewicz H.B., Paulson H.L., McLoughlin H.S. (2017). Evaluation of Antisense Oligonucleotides Targeting ATXN3 in SCA3 Mouse Models. Mol. Ther. Nucleic Acids.

[100] McLoughlin H.S., Moore L.R., Chopra R., Komlo R., McKenzie M., Blumenstein K.G., Zhao H., Kordasiewicz H.B., Shakkottai V.G., Paulson H.L. (2018). Oligonucleotide therapy mitigates disease in spinocerebellar ataxia type 3 mice. Ann. Neurol..

[101] Friedrich J., Kordasiewicz H.B., O’Callaghan B., Handler H.P., Wagener C., Duvick L., Swayze E.E., Rainwater O., Hofstra B., Benneyworth M. (2018). Antisense oligonucleotide-mediated ataxin-1 reduction prolongs survival in SCA1 mice and reveals disease-associated transcriptome profiles. JCI Insight.

[102] Scoles D.R., Meera P., Schneider M.D., Paul S., Dansithong W., Figueroa K.P., Hung G., Rigo F., Bennett C.F., Otis T.S. (2017). Antisense oligonucleotide therapy for spinocerebellar ataxia type 2. Nature.

[103] Niu C., Prakash T.P., Kim A., Quach J.L., Huryn L.A., Yang Y., Lopez E., Jazayeri A., Hung G., Sopher B.L. (2018). Antisense oligonucleotides targeting mutant Ataxin-7 restore visual function in a mouse model of spinocerebellar ataxia type 7. Sci. Transl. Med..

[104] Evers M.M., Pepers B.A., van Deutekom J.C., Mulders S.A., den Dunnen J.T., Aartsma-Rus A., van Ommen G.J., van Roon-Mom W.M. (2011). Targeting several CAG expansion diseases by a single antisense oligonucleotide. PLoS ONE.

[105] Kourkouta E., Weij R., González-Barriga A., Mulder M., Verheul R., Bosgra S., Groenendaal B., Puoliväli J., Toivanen J., van Deutekom J.C.T. (2019). Suppression of Mutant Protein Expression in SCA3 and SCA1 Mice Using a CAG Repeat-Targeting Antisense Oligonucleotide. Mol. Ther. Nucleic Acids.

[106] Maeder M.L., Gersbach C.A. (2016). Genome-editing Technologies for Gene and Cell Therapy. Mol. Ther..

[107] Kc M., Steer C.J. (2019). A new era of gene editing for the treatment of human diseases. Swiss Med. Wkly..

[108] Hsu P.D., Lander E.S., Zhang F. (2014). Development and applications of CRISPR-Cas9 for genome engineering. Cell.

[109] Ouyang S., Xie Y., Xiong Z., Yang Y., Xian Y., Ou Z., Song B., Chen Y., Xie Y., Li H. (2018). CRISPR/Cas9-Targeted Deletion of Polyglutamine in Spinocerebellar Ataxia Type 3-Derived Induced Pluripotent Stem Cells. Stem Cells Dev..

[110] Nishiyama J., Mikuni T., Yasuda R. (2017). Virus-Mediated Genome Editing via Homology-Directed Repair in Mitotic and Postmitotic Cells in Mammalian Brain. Neuron.

[111] Bak R.O., Gomez-Ospina N., Porteus M.H. (2018). Gene Editing on Center Stage. Trends Genet. Tig.

[112] Takata M., Sasaki M.S., Sonoda E., Morrison C., Hashimoto M., Utsumi H., Yamaguchi-Iwai Y., Shinohara A., Takeda S. (1998). Homologous recombination and non-homologous end-joining pathways of DNA double-strand break repair have overlapping roles in the maintenance of chromosomal integrity in vertebrate cells. EMBO J..

[113] Segal D.J., Beerli R.R., Blancafort P., Dreier B., Effertz K., Huber A., Koksch B., Lund C.V., Magnenat L., Valente D. (2003). Evaluation of a modular strategy for the construction of novel polydactyl zinc finger DNA-binding proteins. Biochemistry.

[114] Seligman L.M., Chisholm K.M., Chevalier B.S., Chadsey M.S., Edwards S.T., Savage J.H., Veillet A.L. (2002). Mutations altering the cleavage specificity of a homing endonuclease. Nucleic Acids Res..

[115] Boch J., Scholze H., Schornack S., Landgraf A., Hahn S., Kay S., Lahaye T., Nickstadt A., Bonas U. (2009). Breaking the code of DNA binding specificity of TAL-type III effectors. Science.

[116] Doudna J.A., Charpentier E. (2014). Genome editing. The new frontier of genome engineering with CRISPR-Cas9. Science.

[117] Shin J.W., Kim K.H., Chao M.J., Atwal R.S., Gillis T., MacDonald M.E., Gusella J.F., Lee J.M. (2016). Permanent inactivation of Huntington’s disease mutation by personalized allele-specific CRISPR/Cas9. Hum. Mol. Genet..

[118] Monteys A.M., Ebanks S.A., Keiser M.S., Davidson B.L. (2017). CRISPR/Cas9 Editing of the Mutant Huntingtin Allele In Vitro and In Vivo. Mol. Ther. J. Am. Soc. Gene Ther..

